# Collagen metabolism as a regulator of proline dehydrogenase/proline oxidase-dependent apoptosis/autophagy

**DOI:** 10.1007/s00726-021-02968-y

**Published:** 2021-04-05

**Authors:** Jerzy Palka, Ilona Oscilowska, Lukasz Szoka

**Affiliations:** Department of Medicinal Chemistry, Medical University of Bialystok, Mickiewicza 2D, 15-222 Bialystok, Poland

**Keywords:** Apoptosis, Autophagy, Collagen metabolism, Proline, Proline dehydrogenase/Proline oxidase, Prolidase, Signalling

## Abstract

Recent studies on the regulatory role of amino acids in cell metabolism have focused on the functional significance of proline degradation. The process is catalysed by proline dehydrogenase/proline oxidase (PRODH/POX), a mitochondrial flavin-dependent enzyme converting proline into ∆1-pyrroline-5-carboxylate (P5C). During this process, electrons are transferred to electron transport chain producing ATP for survival or they directly reduce oxygen, producing reactive oxygen species (ROS) inducing apoptosis/autophagy. However, the mechanism for switching survival/apoptosis mode is unknown. Although PRODH/POX activity and energetic metabolism were suggested as an underlying mechanism for the survival/apoptosis switch, proline availability for this enzyme is also important. Proline availability is regulated by prolidase (proline supporting enzyme), collagen biosynthesis (proline utilizing process) and proline synthesis from glutamine, glutamate, α-ketoglutarate (α-KG) and ornithine. Proline availability is dependent on the rate of glycolysis, TCA and urea cycles, proline metabolism, collagen biosynthesis and its degradation. It is well established that proline synthesis enzymes, P5C synthetase and P5C reductase as well as collagen prolyl hydroxylases are up-regulated in most of cancer types and control rates of collagen biosynthesis. Up-regulation of collagen prolyl hydroxylase and its exhaustion of ascorbate and α-KG may compete with DNA and histone demethylases (that require the same cofactors) to influence metabolic epigenetics. This knowledge led us to hypothesize that up-regulation of prolidase and PRODH/POX with inhibition of collagen biosynthesis may represent potential pharmacotherapeutic approach to induce apoptosis or autophagic death in cancer cells. These aspects of proline metabolism are discussed in the review as an approach to understand complex regulatory mechanisms driving PRODH/POX-dependent apoptosis/survival.

## Introduction

**Fig. 1:**
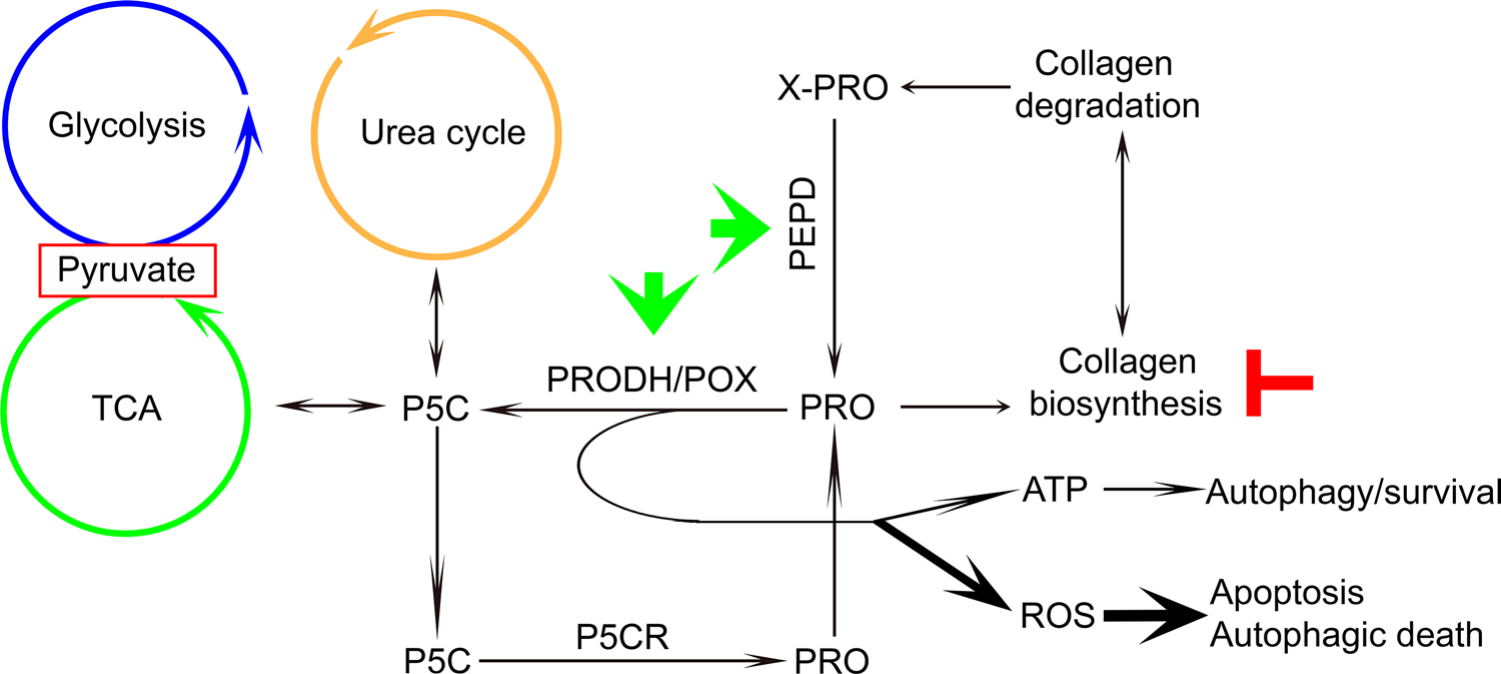
Complex regulatory mechanism for PRODH/POX-dependent apoptosis/survival linking glycolysis, TCA, urea cycles, proline synthesis and degradation with collagen biosynthesis and degradation. Hypothesis is provided that up-regulation of prolidase (PEPD) and PRODH/POX with down-regulation of collagen biosynthesis may represent potential pharmacotherapeutic approach to induce apoptosis or autophagic death in cancer cells. *ATP* adenosine triphosphate, *PEPD* prolidase, *PRO* proline, *PRODH/POX* proline dehydrogenase/oxidase, *PYCR* 1-pyrroline-5-carboxylate reductase, *P5C* 1-pyrroline-5-carboxylate, *ROS* reactive oxygen species, *TCA* tricarboxylic acid cycle

Increasing evidence suggests that proline and collagen metabolism may determine cancer development and progression. Specifically, proline availability determined by collagen biosynthesis/degradation ratio and endogenous synthesis of this amino acid influences redox balance, DNA biosynthesis, epigenetic modifications, apoptosis and autophagy (D’Aniello et al. 2020). Proline degradation by proline dehydrogenase/proline oxidase (PRODH/POX) drives apoptosis, autophagy or survival, depending on the metabolic context. The understanding of the mechanism for the differential PRODH/POX-dependent functions is one of the challenges in cancer cell biology. Proline synthesis enzymes, P5C synthetase and P5C reductase (Ding et al. 2020) as well as collagen prolyl hydroxylases (D’Aniello et al. 2019) are up-regulated in most of cancer types and control rates of collagen biosynthesis. It has been suggested that proline availability and prolyl hydroxylases control progression of cancer cells (Loayza-Puch et al. 2016). Prolyl hydroxylases are Fe^+2^, ascorbate and α-KG-dependent enzymes. They belong to the family of dioxygenases that includes collagen prolyl hydroxylase, DNA and histone demethylases and HIF-1α prolyl hydroxylase (D’Aniello et al. 2019). Since collagen prolyl hydroxylase compete with DNA and histone demethylases for ascorbate and α-KG, collagen biosynthesis may influence metabolic epigenetics. Another member of prolyl hydroxylases (α-KG and succinate dependent) was recently implicated in regulation of HIF-1α transcriptional activity by its targeting for proteasomal degradation (Xiong et al. 2018). Interestingly, proline inhibits HIF-1α prolyl hydroxylase and HIF-1α degradation (Surazynski et al. 2008a). The processes may also affect proline availability for PRODH/POX-dependent functions. The knowledge on functional significance of proline and collagen metabolism in complex metabolic regulatory mechanisms may contribute to understanding differential PRODH/POX-dependent functions (apoptosis/autophagy/survival) in cancer cells (Fig. 1).

### Collagen biosynthesis as a regulator of intracellular proline concentration

Collagen is the most abundant protein in mammals. So far, 29 genetically distinct types of collagen have been described. They differ in terms of their spatial structure, the degree of post-translational modification and the function they perform in various tissues and organs (Ricard-Blum 2011). The most abundant are collagen type I–IV. Their common characteristic feature is the ability to create a trihelical structure composed of identical or different alpha subunits (Brodsky and Ramshaw 1997). The alpha subunits include repeating amino acid triplets containing glycine and often proline and hydroxyproline. The presence of hydroxyproline, an amino acid rarely found in other proteins, is particularly characteristic. It is formed by hydroxylation of prolyl residues in the newly synthesized collagen by prolyl hydroxylase (Myllyharju 2003). It is one of the critical processes in collagen biosynthesis. Any disturbances in this process lead to the inhibition of further stages of collagen biosynthesis contributing to lysosomal degradation of the defective polypeptide chains. It is estimated that about 10–20% of the newly synthesized collagen is degraded intracellularly (Bienkowski 1989). The presence of imino acids, proline and hydroxyproline, constituting about 25% of all collagen amino acids, allows the formation of a triple helix. The trihelical structure allows for the secretion of the molecule outside the cell and makes it resistant to the action of non-specific proteases. However, its function is not limited to tissue support and architecture. It also acts as a ligand for integrin receptors, inducing signalling pathways that regulate cell growth, differentiation and metabolism (Heino 2007). However, the interaction between collagen and integrin receptors is dynamic process. Extracellular collagen is degraded by specific metalloproteinases that cut the molecule into two parts that are further degraded by non-specific proteases (Krane 2008). The resulting short fragments are internalized and degraded intracellularly in the lysosomes to free amino acids, with the exception of imido-dipeptides, e.g., glycyl-proline. Instead, they are degraded by the cytoplasmic prolidase (Surazynski et al. 2008b). It is estimated that in this way the cell recovers about 90% of proline, which can be used for the biosynthesis of new proteins, including collagen (Jackson et al. 1975). However, during last few decades, it has been recognized that proline could play several other functions, e.g., as a stress molecule, regulator of transcription factors and substrate for proline dehydrogenase/proline oxidase (PRODH/POX)-dependent apoptosis/autophagy. Particular attention is focused on the role of proline availability in driving PRODH/POX-dependent functions. In this context, the activity of prolidase (proline supporting enzyme) and collagen biosynthesis (proline utilizing process) could be of critical importance in providing proline for PRODH/POX-induced apoptosis, autophagy or survival.

### PRODH/POX-dependent functions

Proline dehydrogenase (PRODH <, GenBankTM NM_016335) known also as proline oxidase (POX) is flavin-dependent enzyme associated with the inner mitochondrial membrane (Liu and Phang 2012b; Phang et al. 2012). The enzyme catalyses conversion of proline into ∆1-pyrroline-5-carboxylate (P5C). During this process, electrons are transported to electron transport chain producing ATP or they directly reduce oxygen, producing reactive oxygen species (ROS). Therefore, PRODH/POX is considered as a tumor suppressor or pro-survival factor, depending on the environmental conditions (Liu et al. 2012a, 2010; Liu and Phang 2012b; Phang and Liu 2012; Phang et al. 2012). Although the mechanism for switching survival/apoptosis mode is unknown, it seems that PRODH/POX activity/expression could be of importance in these processes. During stress situations (e.g., genotoxic, inflammatory, metabolic as oxygen or glucose deficiency), PRODH/POX is up-regulated in cancer cells and evoke pro-survival activity, both in vitro and in vivo (Mock et al. 1990; Myara et al. 1984; Phang 2019; Zareba et al. 2017). However, up-regulation of PRODH/POX expression in cancer cells is counteracted by inhibitory effect of succinate (TCA metabolite) (Burke et al. 2020; Hancock et al. 2016) and lactate (metabolite of Warburg effect) (Chen et al. 2018) on this enzyme. Importantly, a critical factor that mediate adaptation for such metabolic change is AMP-activated protein kinase AMPK (Laderoute et al. 2006; Wang and Guan 2009). This kinase inhibits energy-consuming processes and activates energy-producing processes to restore energy homeostasis during stress situation for the purpose of survival (Hardie 2008; Kato et al. 2002). Therefore, under glucose deficiency, POX generate ATP. Interestingly, under hypoxia PRODH/POX induces ROS production. However, in such conditions, ROS are not related to apoptosis, but rather to protective autophagy (Liu and Phang 2012b; Phang and Liu 2012). Since mammalian target of rapamycin (mTOR) signalling depends on the availability of nutrients (Reiling and Sabatini 2006), it seems that this pathway is important in POX regulation. In fact, inhibition of mTOR by rapamycin was found to upregulate PRODH/POX (Liu and Phang 2012b; Pandhare et al. 2009). However, in conditions of nutrient availability, silencing of PRODH/POX also induced pro-survival phenotype of cancer cells (Zareba et al. 2017). Therefore, it has been suggested that proline availability for PRODH/POX could differentiate the enzyme-dependent functions.

### The role of proline in cell metabolism and collagen biosynthesis

Studies of last decade provided several lines of evidence for regulatory role of proline availability for PRODH/POX-dependent apoptosis/autophagy in cancer cells (Phang 2019). Large quantity of proline comes from protein degradation. Deregulation of energetic metabolism in cancer cells due to Warburg’s effect facilitates protein degradation as an alternative source of energy. Several studies showed that proline concentration is increased in cancer cells (Catchpole et al. 2011; Hirayama et al. 2009). Both hypoxia (Kakkad et al. 2010) and glucose depletion (Pandhare et al. 2009) were found to induce activity of metalloproteinases, MMP-2 and -9, suggesting the mechanism for the increase in cellular proline concentration. When glucose supply is limited, cancer cells may select proline as an alternative energy source, since proline has an advantage over fatty acids and glutamine, which like glucose requires delivery by the circulation. Therefore, proline may represent energy sense molecule and energy substrate. PRODH/POX expression is often down-regulated in various tumors, limiting mitochondrial proline degradation and PRODH/POX-dependent apoptosis and autophagy. Some studies suggested that critical factor for the process is proline availability that depends on the activity of prolidase (enzyme supporting cytoplasmic proline level) and the rate of proline utilization in process of collagen biosynthesis (Zareba et al. 2020, 2017; Zareba and Palka 2016).

Most of free proline is released from collagen degradation products by cytoplasmic prolidase [E.C.3.4.13.9], known also as Peptidase D or Iminopeptidase (Myara et al. 1984). It cleaves imido-dipeptides with C-terminal proline (Mock et al. 1990). The role of prolidase in recycling proline for collagen biosynthesis is well documented (Laderoute et al. 2006; Miltyk et al. 2005; Surazynski et al. 2008b). The enzyme activity as well as collagen biosynthesis were found to be co-ordinately up-regulated by β1-integrin receptor (Ivaska et al. 1999; Palka and Phang 1997) and insulin-like growth factor (IGF-I) signalling (Miltyk et al. 1998). Increase in the enzyme activity is due to its phosphorylation on serine/threonine residues (Surazynski et al. 2005).

Prolidase plays also important role in regulation of transcription factors. Overexpression of prolidase in colorectal cancer cells contributed to increase in nuclear hypoxia-inducible factor (HIF-1α) level and HIF-1α-dependent gene products, e.g., vascular endothelial growth factor (VEGF) and glucose transporter-1 (Glut-1) (Surazynski et al. 2008a). The activity of HIF-1α is regulated primarily at the level of its degradation (Jaakkola et al. 2001). The hydroxylation of specific proline residues in the oxygen-dependent domain (ODD) by prolyl hydroxylase (PHD) targets HIF-1α for ubiquitination and proteasomal degradation. It has been found that proline inhibits the degradation of HIF-1α, up-regulating its transcriptional activity. Another transcription factor affected by prolidase is NF-kB. Inhibition of prolidase activity by Cbz-pro contributed to up-regulation of NF-kB expression in fibroblasts (Wang and Guan 2009). On the other hand, transfection of colorectal cancer cells with prolidase vector was found to inhibit NF-kB expression (Wang and Guan 2009). The link between collagen biosynthesis and NF-kB is that the transcription factor is a potent inhibitor of gene expression for α1 and α2 subunits of type I collagen (Kouba et al. 1999; Miltyk et al. 2007; Rippe et al. 1999).

PRODH/POX down-regulates HIF-1 α transcriptional activity (Liu et al. 2009a). When proline (inhibitor of HIF-1α degradation) is utilized by PRODH/POX, then HIF-1α undergoes proteasomal degradation limiting its pro-survival functions. PRODH/POX, the only proline degrading enzyme, initiates proline conversion to glutamate and α-ketoglutaric acid that inhibits transcriptional activity of HIF-1, since it is co-substrate of PHD, an enzyme, which hydrolyses specific prolyl residues of HIF-1α. Increase in PHD activity contributes to increase in HIF-1α degradation and a decrease in HIF-1 dependent gene expressions (Liu et al. 2009a; Verma 2006).

The above data suggest that proline plays important role in cellular metabolism. Therefore, collagen biosynthesis, the main utilizer of proline, may represent regulatory mechanism for proline-dependent functions. In fact, collagen biosynthesis is tightly regulated by growth factors, hormones and integrin receptor ligands at both transcriptional and post-transcriptional levels (Sienkiewicz et al. 2004).

Several lines of evidence suggest that inhibition of collagen biosynthesis increases intracellular proline concentrations, making it available as a substrate for PRODH/POX-dependent functions. An example is inhibition of collagen biosynthesis by betulin derivative contributing to PRODH/POX-dependent apoptosis in endometrial adenocarcinoma cells (Szoka et al. 2017). Similar effect on collagen biosynthesis and apoptosis was found in leiomyoma cells treated with 2-methoxyestradiol (Salama et al. 2006) or in adenocarcinoma endometrial cells treated with PPARγ ligands (Fan et al. 2018; Surazynski et al. 2009). The mechanism for PPARγ-dependent inhibition of collagen biosynthesis was found at the level of NF-kB stimulation (Karna et al. 2006). As mentioned above, NF-kB is potent inhibitor of collagen gene expression. However, the PPARγ-induced functions are dependent on the status of estrogen receptor (Bonofiglio et al. 2005; Fan et al. 2018). It has been suggested that ERβ evokes pro-apoptotic effects, while ERα anti-apoptotic (Lu and Katzenellenbogen 2017). Probably, the mechanism of the process is a result of interaction between ERα and PPARγ (Bonofiglio et al. 2005; Kociecka et al. 2010). In view of estrogen-dependent modulation of collagen biosynthesis (Karna et al. 2006; Surazynski et al. 2009), it cannot be excluded that ERα/PPARγ cross-talk regulate proline availability for PRODH/POX-dependent functions.

High cytoplasmic proline concentration could be also considered as a pro-survival and pro-inflammatory since it contributes to up-regulation of transcriptional activity of HIF-1α that induces expression of COX-2, VEGF, TNF-α, TGF-β, IL-1, NF-κB (Surazynski et al. 2008a). Some of the cytokines stimulate collagen biosynthesis (e.g., COX-2, TNF-α, TGF-β) others inhibit the process (e.g., IL-1, NF-κB). Up-regulation of collagen biosynthesis removes proline from cytoplasm, limiting its availability for PRODH/POX-dependent functions. However, it may also contribute to tissue fibrosis, usually accompanying prolonged inflammation in cancer tissues. In view of cancer therapy, severe tissue fibrosis can impair drug delivery to the tissue. This suggests that enhanced collagen biosynthesis is not beneficial both from the point of view of molecular mechanism (POX-dependent functions), tissue function (fibrosis) and pharmacotherapy (impaired drug delivery). Although the complex regulatory mechanisms driving PRODH/POX-dependent functions are not well understood, in general, the above data suggest that inhibition of collagen biosynthesis in cancer cells contributes to increase in PRODH/POX-induced apoptosis and the process (collagen biosynthesis) could be considered a target for cancer treatment.

### Cell metabolic processes regulate proline availability for PRODH/POX-dependent function

PRODH/POX cooperate with P5C reductase (P5CR) participating in proline turnover between mitochondria and cytoplasm. The conversion of proline to P5C that is shuttled between mitochondria and cytosol is coupled to glucose metabolism by pentose phosphate pathway (Pandhare et al. 2009; Phang et al. 2012, 2008). It is vital in maintenance of redox balance in a cell due to participation of NADPH/NADH in conversion of P5C to proline. Moreover, P5C is converted by P5C dehydrogenase (P5CDH) to glutamate, which is a precursor of α-ketoglutaric acid—a component of TCA cycle. PRODH/POX and ornithine aminotransferase (OAT) could also transform proline into ornithine that enters urea cycle (Liu and Phang 2012b). In view of the inhibitory role of PRODH/POX in tumor progression (Donald et al. 2001; Liu et al. 2005; Maxwell and Rivera 2003), all those metabolic cycles are of great importance in the PRODH/POX-dependent functions in neoplastic cells.

The role of interconversion of proline, P5C, ornithine and glutamate in PRODH/POX-dependent functions was described earlier (Huynh et al. 2020; Karna et al. 2020). However, some cells do not express PRODH/POX, e.g., fibroblasts (Downing et al. 1977). The main function of the cells is biosynthesis of extracellular matrix constituents, including collagen. Therefore, PRODH/POX-dependent degradation of proline is undesirable in these cells. Proline derived from protein degradation is involved directly in process of collagen biosynthesis. It has been documented in several cell culture conditions (Karna et al. 2020). However, the process of collagen synthesis requires glutamine metabolism, particularly the synthesis of proline from glutamate and P5C (Hamanaka et al. 2019). During conversion of glutamate into proline, two molecules of NADPH are oxidized, one in mitochondria and one in cytosol (Li and Wu 2018). Probably, it is required for maintenance of redox potential, alternatively proline bearing reducing potential is utilized in collagen synthesis. In this way, collagen biosynthesis and glutamine metabolism is coupled to maintain redox balance. It has been proved in recent studies showing that proline biosynthesis supporting collagen biosynthesis is a vent for TGF-β-induced mitochondrial redox stress (Schworer et al. 2020).

In cancer cells, collagen biosynthesis is not a priority process as in fibroblasts. Probably in cancer cells, collagen biosynthesis modulates proline availability for PRODH/POX. In these cells, proline for collagen biosynthesis comes preferentially from P5C (e.g., generated from glutamine or ornithine). P5C conversion into proline is catalysed by three isoforms of P5C reductase: mitochondrial PYCR1/2 responsible for proline synthesis from glutamate and PYCRL, responsible for proline synthesis from ornithine (De Ingeniis et al. 2012). It seems that intracellular proline derived from collagen degradation at first has to be processed by PRODH/POX yielding P5C in mitochondria (that is mixed with P5C from other sources) which in cytoplasm is converted back to proline by PYRCL. Proline produced from P5C derived from different sources could be a substrate for collagen biosynthesis or, when the process of collagen biosynthesis is inhibited, it could be again degraded by PRODH/POX. The shuttling proline-P5C between mitochondria and cytoplasm, called “proline cycle” (Phang et al. 1981) may work as an engine for regulation of oxidoreductive potential. The intensity of proline cycle depends probably on complex regulatory mechanism for cell metabolism among which, the rate of collagen biosynthesis that utilizes substrate for PRODH/POX seems to play important role. The conversion of P5C to proline requires the presence of NADPH that is oxidized to NADP +. The generation of NADP + is favoured when collagen biosynthesis is increased. The process is coupled with pentose phosphate pathway that utilizes NADP + into NADPH in reaction catalysed by glucose 6-phosphate dehydrogenase, contributing to synthesis of purine nucleotides for DNA biosynthesis. In this context, collagen biosynthesis could reflect DNA biosynthesis. However, when collagen biosynthesis is up-regulated, it decreases proline availability for PRODH/POX, limiting proline cycle. In such a case, P5C is produced from glutamate and ornithine. However, production of proline from ornithine mediates a transfer of reducing potential from cytosolic NADPH into mitochondrial NAD + limiting NADPH pool for P5C reductase and NADP + for glucose 6-phosphate dehydrogenase. Therefore, collagen biosynthesis may play critical role not only in regulation of proline availability for PRODH/POX-dependent functions but also in metabolism of glutamine/glutamate and urea cycle (Huynh et al. 2020).

Based on the above divagations, the question arises what is the role of prolidase (as an enzyme supporting free proline) in regulation of PRODH/POX-dependent functions. It seems that in the mechanism of PRODH/POX-induced apoptosis or survival, proline availability and PRODH/POX expression are equally important. Previously, we have found correlation between PRODH/POX and prolidase in MCF-7 cells (Zareba et al. 2020, 2017). PRODH/POX silencing contributed to drastic increase in prolidase activity, while overexpression of prolidase attenuated PRODH/POX expression contributing in both conditions to pro-survival phenotype of the cells. PRODH/POX silencing was also correlated to collagen biosynthesis inhibition. Although the mechanism of this process requires further studies, it seems that collagen biosynthesis inhibition may result from down-regulation of prolyl hydroxylase, an important enzyme in collagen biosynthesis. It has been documented that free proline inhibits prolyl hydroxylase (Surazynski et al. 2008a) suggesting a mechanism for proline-dependent attenuation of collagen biosynthesis. On the other hand, increase in prolidase activity creates conditions for proline availability for PRODH/POX-dependent functions.

In view of the data, it seems that the extent of PRODH/POX expression may represent another player in complex regulatory processes mediated by proline metabolism. Whether up-regulation of PRODH/POX at proline availability in cancer cells could contribute to apoptosis requires to be explored. Therefore, the mechanisms for regulation of PRODH/POX are of great importance.

### Regulation of PRODH/POX expression

The most potent inducer of PRODH/POX activity is tumor suppressor p53 (Phang et al. 2008; Polyak et al. 1997). Transcriptional regulation of PRODH/POX by p53 was found in the PRODH/POX promoter, containing a p53-response element (Maxwell and Kochevar 2008). PRODH/POX expression is also activated by peroxisome proliferator-activated gamma receptor (PPARγ), ligand-dependent transcription factor (Willson et al. 2000). Also, in this case PPARγ response element (PPRE) was found in the PRODH/POX promoter (Pandhare et al. 2006).

Thiazolidinedione (TZD, as rosiglitazone, troglitazone, and pioglitazone) are well-known agonist of PPARγ (Fumery et al. 2017). They have been also shown to evoke anticancer potential, especially for obesity-related cancers as a prostate, breast, colon, liver, thyroid, lung, and pituitary cancers (Kazberuk et al. 2020; Wang et al. 2017). These properties have been linked to the anti-inflammatory activity of PPARs. PPARγ was found to upregulate PRODH/POX expression (Pandhare et al. 2006), accompanied by ROS generation in several cancer cell lines (Kim et al. 2007; Liu and Phang 2012a; Pandhare et al. 2006; Wang et al. 2011). PPARγ-dependent up-regulation of PRODH/POX expression was linked to inhibition of tumour necrosis factor α (TNF-α) as well as interleukin 1β (IL-1β) (Han et al. 2017). Several studies have also suggested a relationship between PRODH/POX and cyclooxygenase II activity (COX-2), showing that high expression of PRODH/POX inhibited COX-2 expression (Liu et al. 2009b). The up-regulation of COX-2 has been found in many cancers and correlated with a worse prognosis in several types of malignant neoplasms (Coussens and Werb 2002). The anticancer potential of non-steroidal anti-inflammatory drugs (NSAIDs) is well-known phenomenon (Fujii et al. 2014; Harris et al. 2012; Toloczko-Iwaniuk et al. 2020). Moreover, NSAIDs are ligands of PPARγ (Kazberuk et al. 2020; Seetha et al. 2020). However, the mechanism of anticancer activity of NSAIDs is still unknown.

**Fig. 2:**
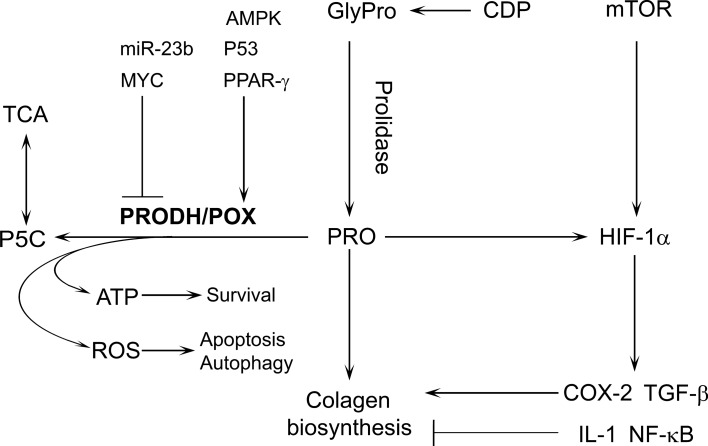
Relationship between prolidase and PRODH/POX expressions, collagen biosynthesis and degradation, and transcriptional activity of HIF1-α in the regulation of PRODH/POX-dependent apoptosis, autophagy and survival. *AMPK* AMP-activated protein kinase, *ATP* adenosine triphosphate, *CDP* collagen degradation protein, *COX*-2 cyclooxygenase 2, *GlyPro* glycyl-proline, *HIF-1α* hypoxia-inducible factor 1-alpha, *IL-1* interleukin 1, *mir-23b* non-coding small RNA regulating PRODH/POX translation and PRODH/POX gene expression, *mTOR* mammalian target of rapamycin, *MYC* genes and proto-oncogenes regulator, *NF-kB* nuclear factor kappa-light-chain-enhancer of activated B cells, *P53* tumor suppressor protein 53, *P5C* D1-pyrroline-5-carboxylate, *PPAR-g* peroxisome proliferator-activated gamma receptor, *PRO* proline, *PRODH/POX* proline dehydrogenase/proline oxidase, *ROS* reactive oxygen species, *TGF-β* transforming growth factor β, *TCA* tricarboxylic acid cycle

Knocking down PRODH/POX inhibited the apoptotic response to PPARγ ligands (Liu et al. 2012b; Phang et al. 2008). Therefore, it was suggested that TZD-dependent apoptosis undergoes through PRODH/POX-dependent ROS generation. Among the most potent down-regulators of PRODH/POX is miR-23b* (Liu et al. 2010) and oncogenic transcription factor c-MYC (Liu et al. 2012b). Overexpression of miR-23b* attenuated PRODH/POX activity, ROS generation, apoptosis and augmented HIF-1 signalling (Liu et al. 2010; Surazynski et al. 2008a) contributing to oncogenesis and tumor progression. Of great importance is finding that MYC inhibited PRODH/POX expression through up-regulation of miR-23b* attenuating production of ROS and apoptosis (Liu et al. 2012b). The factors involved in this process are outlined in Fig. 2

## Concluding remark

The above data suggest that collagen metabolism (synthesis and degradation) through modulation of proline availability for PRODH/POX is involved in regulation of metabolism of glutamine, TCA and Urea cycles. Since collagen biosynthesis and more specifically, collagen prolyl hydroxylase competes with DNA and histone demethylases for ascorbate and α-KG, the inhibition of collagen biosynthesis could attenuate epigenetically cancer progression, while proline (saved in this process) could serve as an inhibitor of prolyl hydroxylase and substrate for PRODH/POX-dependent apoptosis. These findings allow to provide hypothesis that up-regulation of prolidase and PRODH/POX with down-regulation of collagen biosynthesis may represent potential pharmacotherapeutic approach to induce apoptosis or autophagic death in cancer cells. Such a possibility is currently under investigation.
